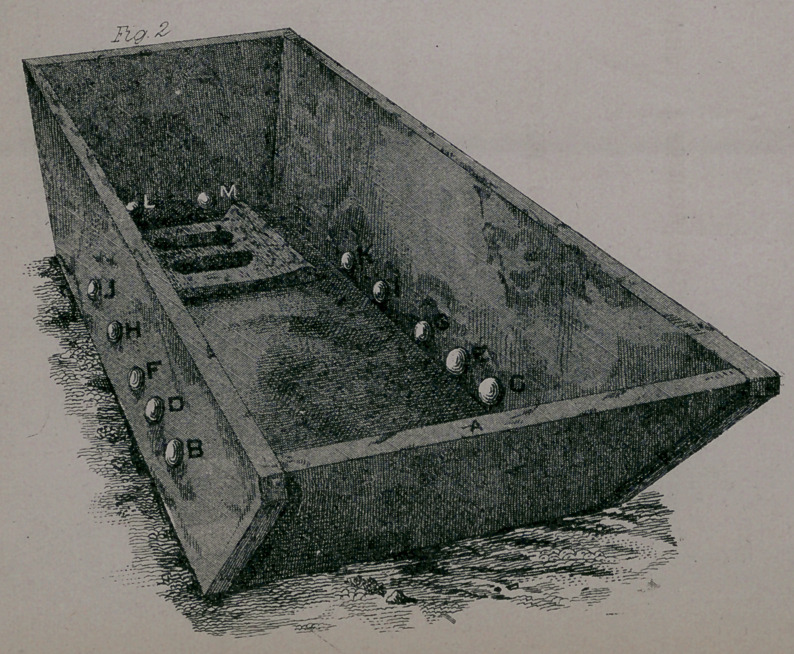# Therapeutics of the Electro-Thermal Bath; Embracing the Use of Induced and Galvanic Currents in Such a Bath

**Published:** 1873-11

**Authors:** Justin Hayes

**Affiliations:** Chicago


A MONTHLY RECORD OF

*Medicine, Surgery and the Collateral Sciences.*

EDITED BY J. ADAMS ALLEN, M.D., LL.D.; AND WALTER HAY, M.D.

---

VOL. XXX. — NOVEMBER, 1873. — NO. 11.

---

### Original Communications.

ARTICLE I.—*Therapeutics of the Electro-Thermal Bath; Embracing the Use of Induced and Galvanic Currents in such a Bath.* By JUSTIN HAYES, M.D., Chicago.

In the lithographs, Fig. 1 represents the helix and key-board, and Fig. 2 the electro-thermal bath tub. The letters by the switches on the key-board, and electrodes in the tub, are arranged alphabetically, except the switches Q and R, which represent the off currents, and N, which connects the battery current with the helix. The switch A is connected by a wire to the head-plate A in the tub; the switches B C are connected with the B C electrodes in the tub; and so on in alphabetical order till you reach the foot electrodes L and M. O represents the commutator or current-changer; P the rheotome, and S the electro-magnet or core. The head-plate, or electrode, is a piece of sheet copper, the form oblong, and breadth seven inches; this is attached to the centre of the head of the tub, and covered by a perforated board; over this is placed a rubber cloth insulator, to protect the spine from the direct action of the current; this insulator may be narrowed at pleasure by rolling it on the sides, so the current may approach still nearer to the spinal cord when the condition of the patient indicates it; in cases where the current should be applied directly to the spine, while lying in the bath, it should be removed.

GENERAL CURRENTS IN THE ELECTRO-THERMAL BATH.

These currents are termed *general* because they are given off from so many electrodes, consequently exerting a general influence on the whole system, tranquilizing and producing tonic, and stimulating; or sedative and depressing effects, according to the strength and duration of the applied currents. The switches on Fig. 1 are set for the general currents; these are the currents that are used when the patient is first placed in the bath, and are switched on before the patient comes out, let the other treatment during the time they are in the bath be as varied as it may be. The head-plate is positive, and all the other electrodes in the tub are negative. In cases where the negative bath is required in the beginning of the treatment (which is the exception), raise the handle of the commutator; this reverses the currents, making the head-plate negative and the other electrodes positive. To make it a galvanic bath, insulate one arm of the commutator by slipping a piece of paper under it, and connect the galvanic battery to the key-board, having another commutator and current selector for this current, and you are enabled to make all the changes in directing this current, that you are with the induced current in the electro-thermal bath first mentioned. The galvanic current should be so connected with the cells of the battery, that you may add from one to three elements at a time, till you have the desired amount. It is also quite necessary to have the current connected with a galvanometer, that you may know the strength of the current, when it is conducted to the bath; and when treating very sensitive patients the rheostat should be connected, for this current is one that will do great good or great harm, according to its use; and the physician should always know the amount that he is using, and closely observe the immediate effect upon his patient, before leaving him in the hands of the operator. The two coils, which enter into the construction of the faradaic apparatus, from which the induced currents for the electro-thermal bath are obtained, are composed of thick and short wire, which give a quantity rather than intensity current. The helix and key-board represented in Fig. 1 were made to the order of the late Dr. Young, of Cleveland, Ohio, with the exception of an increased number of keys, which I have added to meet the improvements I have made in the tub electrodes, (which improvements are offered to the profession free of charge.) Dr. Young patented his invention, and it is now held by Dr. John C. Mairs, Steubenville, Ohio.

Twelve years ago, while residing in Cleveland, Ohio, I was invited by Dr. Young to observe the effects of the electro-thermal bath on his patients, which I did occasionally for about six months. Although his theory as to the bath "reproducing diseases" was erroneous, and his treatment hap-hazard, I became convinced that his invention was an advance over anything of the kind that I had a knowledge of, and that its efficacy in treating diseases that the bath was appropriate for, might prove a great success when properly prescribed and used by scientific physicians. I purchased two baths and the apparatus pertaining thereto, which were somewhat similar, and after using them for a few months I discarded one, and have made my improvements from the one previously mentioned.

As there has been no scientific work or essay on the use of the electro-thermal bath, (embracing the use of induced and galvanic currents in such a bath,) I desire to place some of my experience before the profession. At the same time I wish to be understood that in my practice I have ever eschewed hobby-riding, and sought the best means of the profession for my patients.

To prepare the electro-thermal bath for a patient, it is necessary to know if the patient is very sensitive to warm or cool water; the bath should be of a temperature to meet the feelings of the patient pleasurably on first going into it. As a general rule it should be prepared at 98° Fahrenheit, and gradually increased till it reaches in some extreme cases 110°; the guide for increasing the temperature must be the patient's feelings, as no set rule can be given for every case. The time in the bath ranges from eight to thirty minutes; the first one taken should not be more than ten or twelve minutes; the effects of this bath will serve as a guide to the time occupied in subsequent treatment of the case. The patient lies down in the bath, being covered at the same time with a canton flannel sheet, the head resting on a sponge which is under or on napkins folded above the insulator, the feet resting against the foot of the tub, unless the patient is too short to reach it, then they should be supported by a foot-adjuster; a small napkin wet in cold water, wrung out so as not to drip, should be placed on the forehead; then turn the switch N on to the post, as shown in Fig. 1; this sends the currents at once through the water to the patient. Before connecting the current to the helix, be sure that the core is nearly out of the coil, so that the patient will not receive too much of the current in the beginning, and, to make this doubly sure, when through treating a patient, disconnect switch A, as well as N, so that when you begin to treat the next patient, should you forget to withdraw the core in starting the machine, the patient will receive no electricity till you connect the head-plate by the switch A, and this will be very sure to call the attention of the operator to the position of the core. As soon as the currents are conducted to the patient, the operator should at once commence dipping with a handled dipper which holds about one quart, pouring the water gently on the upper part of the chest, beginning on the opposite side of the patient, and bringing it around to the side next the front of the tub; this is continued more or less during the treatment. Increase the temperature of the water from the beginning, by a small stream till the patient feels warm enough; push the core in to give the current intensity enough to be perceptible to the patient. These currents should be continued from three to five minutes, then take the electrode connected with the positive off-current R, place it on a soft wet sponge, turn the sheet down to the hips, throw off switch A, grasp the sponge firmly in the hand, and apply the back of the fingers over the region of the liver—that the operator may feel the strength of the current, and test the sensibility of the patient to its action; if it gives no pain, apply the sponge, pressing it gently, carrying it over the spleen, then downwards in the course of the descending colon to the left iliac fossa, passing across the lower part of the abdomen to the right iliac fossa, and from here up the course of the ascending colon, continuing over the transverse colon and the small intestines; then apply it, as in the beginning, over the region of the liver, repeating the manipulation three or four times; then spread the sheet back over the chest, switch on A, as in the beginning of the treatment, and proceed as at first, until one minute before the expiration of the time; then place your hand under the neck to assist the patient in sitting up in the tub; throw off switch A; take the electrode connected with the positive off-current R, place it in the sponge, and press the sponge on the nape of the neck a little one side of the spine, carrying it down the whole length of the back, alternating from side to side from three to five times, carrying it *near* the spine but not directly on it. This finishes the bath, given in general debility.

The patient should be well dried off, and afterwards rubbed with the dry hand if there is any chilliness. A small handful of salt is usually thrown into the bath, unless iron is indicated in the case; then use from one to two ounces of the following solution:

R. Ferri Sulphatis, . . . . . . oz. j.
Acidi Sulphurici, . . . . . oz. iij.
Aquæ puræ, . . . . . . . oz. viij.
M.
S. Use in each bath from one to two ounces.

Seven years ago, in giving the iron bath, I used from one to two drachms ferri sulphatis, making the bath turbid, and staining everything it came in contact with; thus making it necessary for the patient to take another bath of clear water to free the surface of the body from the iron stains. At that time, Mr. Varley, of London, the electrician of the Atlantic Cable Co., was receiving my treatment. One of the remedies prescribed was the electrical iron bath; when he learned how it was prepared, he said that in his experiments he had discovered how to make a water solution of the ferri sulphatis clear, by adding acidi sulphurici, so that it would not stain a piece of white linen when dipped in it. From his suggestion the above formula was made.

Mr. E——, a resident of Chicago, came to me in July, 1871, for treatment. He was about forty years of age, of good habits, and was suffering from general debility without any apparent cause; he was not overtaxed in any way, as his business required no more of his time than he chose to give it—the pulse feeble, about sixty-four per minute; tongue slightly furred; did not suffer pain; complained of nothing but weariness and want of ambition, and felt worse about 11 A. M., showing a slight climatical periodicity; had a poor appetite most of the time, when he did relish his food and eat to satiety felt heavy and less disposition to exercise; bowels torpid, and skin inclined to be dry; urine normal, but a little less in quantity than when in health; slept well the first of the night, but restless and uneasy the latter part of it. I prescribed the electro-thermal bath with iron, as previously directed for cases of general debility; time in bath ten minutes; advised him to take another the next day, which he did; he slept a little better and was not quite as stupid at 11 A. M. I prescribed, in connection with this treatment, the following:

R. Quiniæ Sulphatis, . . . . . gr. xij.

Piperinæ, . . . . . . gr. iiij.

M. Fiat pulv. No. xij.

S. Take one at meals and bedtime.

Advised him to come in the day following for treatment. On his return the third day he said he felt but little better, and feared it was not the right treatment for him—was afraid the warm water would make him weak. I said to him that I was confident if he would continue treatment for eight or ten days, he would be pretty well. I prescribed the foot-bath with the *vitalized* treatment, to be alternated with the electro-thermal bath, as previously given. The vitalized treatment was given, as follows: His feet were placed in a foot-bath at 98° Fahrenheit, and gradually raised to 101° with the negative electrode in the water, the positive electrode in a soft sponge and placed by the operator over the region of the liver—with an intensity current appreciable to the patient—from here it was passed over the right pectoralis major muscle, across the chest to the left pectoralis major muscle, down the side over the spleen to the left iliac fossa, across the lower part of the abdomen to the right iliac fossa, up the ascending colon and over the transverse, finishing the circuit over the small intestines. This was repeated twice, then the sponge was grasped in the left hand of the operator, the right hand carried slowly over the chest and abdomen, following the same course as the sponge, for four or five times. Then the sponge with the positive electrode was placed on the nape of the neck, one side of the spine and carried down the whole length of the spine, alternating twice from side to side. This was followed by using the hand for an electrode as in treating the chest and abdomen, carrying the hand over the spine, as well as at each side. The treatment was concluded by the patient holding the sponge with the positive electrode for three minutes—

time of treatment being twelve minutes. The tenth day he was so well that he required no more treatment.

The following cases will show the treatment for arresting the growth of fibrous tumors of the uterus:

CASE I. Mrs. E——, aged forty-two years—from the central part of the State; came for treatment in the spring of 1867 (the exact date I am unable to give, being destroyed in the fire, Oct. 9th, 1871), suffering with intra-mural fibrous tumor, and menorrhagia. Upon examination, I found the case to be a very serious one, and advised her husband to get a physician who had experience in the treatment of ladies, for counsel. Dr. DeLaskie Miller was called; his diagnosis concurred with mine as to the nature of the tumor. He thought her general health might be improved—had but little, if any, encouragement to give as to removing the tumor. As to the use of electricity in such cases he had had no experience; suggested the use of iodine externally over the abdomen, and iodide and bromide potassa internally; which was given according to the following formula:

| | | |
|---|---|---|
| R. | Potassii Iodidi, }<br>Potassii Bromidi, } . . . | *aa* dr. vj. |
| | Syr. Sarsaparillæ Comp., . . . | oz. viij. |
| M. | | |
| S. | Take one teaspoonful three times a day. | |

Upon examining the uterus, the os was found in an irregular nodulated condition, and within one inch of the labia majora, the os tincæ opened so that it would admit the index finger, three-quarters of an inch; the uterus was about the size of a quart bowl, and irregular in form. In the year 1848, she commenced having profuse hemorrhage at her menstrual period, lasting from one to three weeks; this continued up to the time she came to consult me. Some of her physicians had ascertained that she had a tumor, and within four or five years previous to her coming to me, she could both see and feel the enlargement above the pubis. She was very much emaciated, exhausted and mentally depressed; and as she never had received any permanent benefit from her physicians, did not expect it now. The treatment was commenced by applying iodine over the abdomen, then taking the electro-thermal bath with iron, commencing with the currents as recommended in general debility; a vaginal injection of tepid water, as soon as she was comfortable in the bath, this followed by a bromine wash, according to the following formula :

R. Bromine, . . . . . . . gtt. viij.
Aqua Dist., . . . . . . oz. iiij.

M.

S. Use one teaspoonful in two tablespoonsful of tepid water once a day.

After taking the general currents three minutes, having the temperature properly increased, all the switches that carried the currents to the tub were disconnected except I; then H was connected to the upper post, making a concentrated transverse current through the abdomen to the tumor; changing the direction of the currents, by changing the commutator, alternating from positive to negative, and negative to positive, four or five times; with an intensity current that caused a perceptible motion of the abdomen from side to side, when the current was changed; this was continued two minutes, then H was disconnected from the upper post, the operator taking the positive off-current R, placing the electrode in a sponge, pressing it firmly over the tumor on the opposite side of the abdomen from the I electrode, which is connected with the I switch; changing the commutator four or five times; then disconnecting I and connecting J and K, as shown in Fig. 1, placing the sponge with the positive electrode on the abdomen over the upper part of the tumor; this was continued two minutes; then the switches were connected for the general currents as in Fig. 1, these were continued two minutes, and the treatment was concluded by sponging the back as previously directed. This treatment was repeated daily for one week, when her menses appeared; they were less than usual at the commencement, and the hemorrhage was much less; the fifth day I placed a tampon of cotton wool containing ferri subsulphatis, gr. iij, zinci sulphatis, dried, gr. ij, and introduced it as far up as I could in the os uteri. The sixth day there was very little hemorrhage; the seventh she was able to resume the former treatment, which was continued till her menses appeared again; at this time they were nearly normal, and she was not confined to her room at all during this period. The flow ceased the sixth day from commencement. On the fifth day I prescribed the vitalized treatment with foot-bath as previously described, with this exception: the positive electrode was in the foot-bath and the negative in the hand of the operator, until the last three minutes of the treatment, when the negative electrode was placed in the hands of the patient; this was continued two minutes when the current was reversed and this continued one minute; this concluded the treatment; the following day it was repeated; the seventh day she resumed again the former treatment.

She was under treatment eight weeks, when she left for home; promising to return if she was worse. The uterus was diminished about one-third in size and her general health very much improved. She returned four or five months afterwards. I then made an examination and found the tumor still smaller than when she left; as she was feeling quite well she wished to remain only for a few days' treatment. I heard from her a few months since; then she was attending to her household duties and felt no inconvenience from her former trouble.

(To be continued.)

## Figures and Tables

**Fig. 1 f1:**
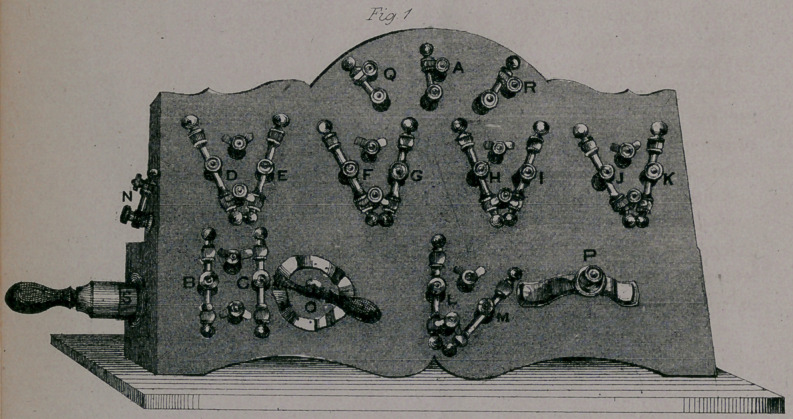


**Fig. 2 f2:**